# Placental mitochondrial DNA content is associated with childhood intelligence

**DOI:** 10.1186/s12967-019-2105-y

**Published:** 2019-11-08

**Authors:** Esmée M. Bijnens, Catherine Derom, Steven Weyers, Bram G. Janssen, Evert Thiery, Tim S. Nawrot

**Affiliations:** 1 Centre for Environmental Sciences, Hasselt University, Agoralaan Building D, 3590 Diepenbeek, Belgium; 2 Department of Obstetrics and Gynaecology, Ghent University Hospital, Corneel Heymanslaan 10, 9000 Ghent, Belgium; 3 Centre of Human Genetics, University Hospitals Leuven, Herestraat 49, 3000 Leuven, Belgium; 4 Department of Neurology, Ghent University Hospital, Corneel Heymanslaan 10, 9000 Ghent, Belgium; 5 Department of Public Health & Primary Care, Leuven University, Kapucijnenvoer 35, 3000 Leuven, Belgium

**Keywords:** Mitochondrial DNA content, Placenta, Intelligence, Childhood, Twins, DOHaD

## Abstract

**Background:**

Developmental processes in the placenta and the fetal brain are shaped by the similar biological signals. Evidence accumulates that adaptive responses of the placenta may influence central nervous system development. We hypothesize that placental mtDNA content at birth is associated with intelligence in childhood. In addition, we investigate if intra-pair differences in mtDNA content are associated with intra-pair differences in intelligence.

**Methods:**

Relative mtDNA content was measured using qPCR in placental tissue of 375 children of the East Flanders Prospective Twin Survey. Intelligence was assessed with the Wechsler Intelligence Scale for Children-Revised (WISC-R) between 8 and 15 years old. We accounted for sex, gestational age, birth weight, birth year, zygosity and chorionicity, cord insertion, age at measurement, indicators of socioeconomic status, smoking during pregnancy, and urban environment.

**Results:**

In multivariable adjusted mixed modelling analysis, each doubling in placental mtDNA content was associated with 2.0 points (95% CI 0.02 to 3.9; p = 0.05) higher total and 2.3 points (95% CI 0.2 to 4.3; p = 0.03) higher performance IQ in childhood. We observed no association between mtDNA content and verbal intelligence. Intra-pair differences in mtDNA content and IQ were significantly (p = 0.01) correlated in monozygotic-monochorionic twin pairs, showing that the twin with the highest mtDNA content was 1.9 times more likely (p = 0.05) to have the highest IQ. This was not observed in dichorionic twin pairs.

**Conclusions:**

We provide the first evidence that placental mtDNA content is associated with childhood intelligence. This emphasizes the importance of placental mitochondrial function during in utero life on fetal brain development with long-lasting consequences.

## Background

Studies suggest that the placenta, apart from transport of maternal nutrients, growth factors and hormones, also plays a pivotal role in central nervous development through adaptive responses to the maternal environment [1–3]. A suboptimal placental nutrient supply can influence the fine structure of neuronal networks in the brain [4], and therefore limiting final cognitive performance to a level below genetic potential [5]. Mitochondria are intracellular organelles and their main role is to generate energy for the cell. Since metabolic energy is important in the brain at the synaptic level for normal neural communication [6], mitochondria are essential for human cognition [7]. Therefore the maintenance of mitochondrial DNA (mtDNA) content is important to preserve mitochondrial function, cell growth and morphology [8]. Fetuses adapt their mitochondrial structure and metabolism when the supply of nutrients is limited [9] or due to exposure to environmental pollution [10].

It was demonstrated that human ageing entails a decline in mtDNA copy number (mtDNA content) among Danish twins and singletons (18–93 years of age) [11]. Moreover, subjects with low mtDNA copy number in blood had poorer outcomes in terms of physical strength, self-rated health, higher all-cause mortality and also cognitive performance, compared with subjects with high mtDNA copy number [11]. In elderly, a higher blood mtDNA copy number was consistently associated with higher cognitive performance [11, 12].

The Developmental Origins of Health and Disease (DOHaD) hypothesis, often called the Barker hypothesis, states that adverse influences of the early-life environment can result in permanent changes in adulthood [13]. In this regard, twin studies allow estimation of the importance of the individual fetoplacental environment unique to each fetus, while controlling for shared factors, such as maternal environment. Both individuals of a twin pair share the same maternal environment and, in case of monozygotic twins, share the same fetal genes. Each fetus has its own fetoplacental environment, which may differ substantially from that of its co-twin [14]. In the context of fetal programming on cognitive outcomes, we studied placental mtDNA content at birth and intelligence in childhood in a study conducted in twins. Furthermore, we examined the association between placental mtDNA content and child problem behaviour. We hypothesize that placental mtDNA content at birth is associated with intelligence in childhood. In addition, we investigate if intra-pair differences in mtDNA content are associated with intra-pair differences in intelligence.

## Materials and methods

### Subject recruitment

The East Flanders Prospective Twin Survey (EFPTS) is a population based register of multiple births in the province of East-Flanders (Belgium) since 1964 [15]. 376 twin pairs born between 1982 and 1992 were invited, in 1992 and between 1996 and 1999 child IQ was assessed with use of the WISC-R (Wechsler Intelligence Scale for Children-Revised) test [16, 17]. The parents filled out the Child Behaviour Checklist (CBCL) [16]. Of the 376 twin pairs with IQ assessment, we selected those (n = 405 individuals) of which we had placental samples and information about residential history. We excluded 30 participants from our analysis because DNA quality or concentration was insufficient (n = 4), because triplicate measurements of mtDNA content were too variable (difference in quantification cycle more than 0.30) (n = 18) or missing data (n = 8). The number of children included in our analysis was 375. Informed consent was obtained from all participants, and ethical approval was given by the Ethics Committee of University Hospital Ghent and Hasselt University (Registration number: B670201730788).

Data recorded by the obstetrician at birth included gestational age, birth weight and sex of the twins. Gestational age was calculated as the number of completed weeks of pregnancy and was estimated on the basis of last menstrual period combined with real-time ultrasonography in early pregnancy. At time of birth, placentas were examined within 24 h after delivery by a trained midwife following a standardized protocol [18]. Cord insertion was categorized into two groups: central insertion (central, paracentral, paramarginal) and peripheral insertion (marginal, membrane septum and membrane peripheral). For each individual, one placental biopsy was taken close to the surface near the insertion of the umbilical cord, also in twins sharing one placenta, and stored at − 20 °C in a biobank. Zygosity was determined by sequential analysis based on sex, choriontype, blood group determined on umbilical cord blood, placental alkaline phosphatase, and, since 1982, DNA fingerprints [19]. After DNA-fingerprinting, a zygosity probability of 99.9% was reached.

Further we collected information on parental education. Educational level as a proxy of socioeconomic status was categorized into three groups according to the Belgian education system: [1] no education or primary school, [2] lower secondary education and [3] higher secondary education or tertiary education.

Residential addresses of the mothers at birth were geocoded. Urban areas in a 5000 m radius from address was estimated based on Corine land cover 1990 (European Environment Agency). In addition to the individual levels of social economic class indicators, we gathered information on neighbourhood socioeconomic status. Based on their home address, all mothers were assigned to statistical sectors (average area = 1.55 km^2^), the smallest administrative entity for which statistical data are produced by the Belgian National Institute of Statistics (NIS). Belgian census data (FOD Economie/DG Statistiek) derived from the NIS were used to define neighbourhood household income based on annual household income in the year 1994.

### Assessment of neurodevelopment and behaviour during childhood

The neurodevelopment outcome was assessed by the Wechsler Intelligence Scale for Children-Revised (WISC-R). The WISC-R consists of six verbal and six performance subscales and has been validated for use in this population [20]. The verbal subscales are Information (INF), Similarities (SIM), Arithmetic (ARI), Vocabulary (VOC), Comprehension (COM) and Digit Span (DS). The performance subscales are Picture Completion (PC), Picture Arrangement (PA), Block Design (BD), Object Assembly (OA), Coding (COD) and Mazes (MAZ). The scores on the subscales are standardized for age and added up to Verbal (VIQ), Performance (PIQ) and Total Intelligence Quotients (TIQ). In this study, the total scores of the subscales and the TIQ score were analysed. The minimum and maximum scores that can be obtained for total, verbal and performance IQ are 50 and 150 points.

Neurobehaviour was assessed by the Achenbach Child Behaviour Checklist (CBCL). This checklist was developed by Achenbach (1991) to examine the extent to which children have behavioural and emotional problem as perceived by their parents [21]. Although the CBCL allows for the calculation of separate scores corresponding to several behavioural dimensions, in this study we examined the total problem score, and the externalizing and internalizing subdomains.

### Measurement of mtDNA content

DNA was isolated from placental tissue using the QIAamp DNeasy blood and tissue kit (Qiagen, Venlo, The Netherlands). Quality and concentration of the isolated placental DNA was assessed using the Nanodrop 1000 spectrophotometer (Isogen Life Science, Belgium) and the Quant-iT™ PicoGreen dsDNA Assay Kit (Life Technologies, United States) using the Omega Fluostar plate reader.

The method for measuring mtDNA content was described previously [10, 22]. Relative mtDNA content was measured in placental tissue using a quantitative real-time polymerase chain reaction (qPCR) assay by determining the ratio of two mitochondrial gene copy numbers *MTF3212/R3319* and *MT*-*ND1* to a single-copy nuclear control gene *RPLP0*. Reaction mixture and PCR cycles used are given in Additional file 1. Each PCR reaction was carried out in triplicate and three non-template controls as well as six inter-run calibrators were included on each 384-well plate. All samples were analyzed with the 7900HT Fast Real-Time PCR system (Life Technologies).

After thermal cycling, raw data was collected and processed. C_T_ (cycle threshold)–values of the two mitochondrial genes were normalized relative to a nuclear reference gene according to the qBase software (Biogazelle, Zwijnaarde, Belgium). The program uses modified software based on the classic comparative C_T_ method, it takes the reference gene into account and uses inter-run calibration algorithms to correct for run-to-run differences [23]. The coefficient of variation for the mtDNA content in inter-run samples was 5.1%. All samples were analyzed in triplicate and included in the study when the difference in quantification cycle (Cq) value was < 0.30.

### Statistical analysis

For data management and statistical analyses, we used SAS software, version 9.4 (SAS Institute, Cary, NC). All reported p values are two-sided and were considered statistically significant when p < 0.05. The distribution of all variables was inspected. To improve normal distribution, mtDNA content was log_10_-transformed. Mixed modelling was performed to investigate intelligence in association with placental mtDNA content. The twins were analyzed as individuals in a multilevel regression analysis to account for relatedness between twin members by adding a random intercept to the model. The variance–covariance structure was allowed to differ between the three zygosity and chorionicity groups. Mixed modelling was performed adjusted for covariates selected a priori, namely sex, birth weight, gestational age, birth year, zygosity and chorionicity, cord insertion, age at measurement, parental educational level, neighbourhood household income, maternal smoking during pregnancy and urban environment. Mixed models were also used to study the association between child behaviour and placental mtDNA content, adjusting for previous mentioned covariates.

Subsequently, intra-pair differences in mtDNA content were calculated. These intra-pair differences were correlated with the intra-pair difference in total, verbal and performance IQ. Furthermore, the probability of the twin with the highest mtDNA content having the highest IQ in comparison with their co-twin was investigated using a Chi-square test. The analysis was stratified for zygosity and chorionicity, resulting in three groups; dizygotic, monozygotic-dichorionic and monozygotic-monochorionic twin pairs.

## Results

### Characteristics of the study population

Table 1 summarizes the characteristics of the study population including 201 mothers and the 375 children: 93.0% (n = 348) of the participants included both twins from each twin pair, whereas the remaining 7.0% (n = 27) only had one participating twin from each twin pair. Overall, most parents (56.7%) were highly educated. Among the mothers, 8.0% (n = 16) continued smoking during pregnancy. The twin population comprises 194 (51.7%) males and 181 (48.3%) females. The twins had a mean (± SD) birth weight of 2525 ± 514 g, an overall mean gestational age of 36.8 weeks (range 29–42) and 50% was born preterm. Our analysis included 56.5% dizygotic twins, 19.5% monozygotic-dichorionic twins and 24.0% monozygotic-monochorionic twins. The mean (± SD) relative placental mtDNA content was lower in the monozygotic-monochorionic twins (0.98 ± 0.47) compared with the dizygotic-dichorionic twins (1.13 ± 0.55, *p *= 0.03) and monozygotic-dichorionic twins (1.13 ± 0.51, *p *= 0.05). This is shown in Table 2. The twins completed the Wechsler Intelligence Scale for Children-Revised test at a mean age of 11.5 years old (between the ages of 8 and 15 years). Their averaged scores (± SD) were 106.0 ± 15.1 for total IQ, 106.6 ± 14.6 for verbal IQ and 103.9 ± 15.3 for the performance IQ.

**Table 1:** Study population characteristics

*Parental characteristics*	*(n = 201)*
Education	
Low	16 (8.0)
Middle	71 (35.3)
High	114 (56.7)
Neighbourhood income, euro	18,483 ± 2474
Smoking during pregnancy	16 (8.0)
Cigarettes/day during pregnancy	1.5 ± 3.7
Urban environment within 5 km, %	32 ± 12
*Characteristics of twins*	*(n = 375)*
*Birth*	
Birth weight (g)	2525 ± 514
Gestational age, weeks	36.8 ± 2.3
Birth year	1986 ± 2.5
Sex	
Male–male	143 (38.1)
Female–female	131 (34.9)
Male–female	101 (26.9)
Zygosity–chorionicity	
Dizygotic-dichorionic	212 (56.5)
Monozygotic-dichorionic	73 (19.5)
Monozygotic-monochorionic	90 (24.0)
Twins in final study	
Twin with 1 individual in study	27 (7.0)
Twin with 2 individuals in study	348 (93.0)
*Childhood*	
Age	11.5 ± 1.6
Intelligence score (IQ)	
Total IQ	106.0 ± 15.1
Verbal	106.6 ± 14.6
Performance	103.9 ± 15.3

Data presented are mean ± standard deviation or number (percentage)

**Table 2:** Relative placental mtDNA content according to the different zygosity–chorionicity groups

Relative placental mtDNA content	Mean	Standard deviation	p-value (compared to MZMC)
Dizygotic-dichorionic	1.13	0.55	0.03
Monozygotic-dichorionic	1.13	0.51	0.05
Monozygotic-monochorionic	0.98	0.47	–

### Determinants of childhood intelligence

Table 3 shows the covariates associated with the total, verbal and performance intelligence quotient. The multivariable model is a mixed model taking the relatedness between twin member into account and is adjusting for sex, birth weight, gestational age, birth year, zygosity and chorionicity, cord insertion, age at measurement, parental educational level, neighbourhood household income, maternal smoking during pregnancy and urban environment. Total, verbal and performance IQ were higher in descendants from higher educated parents, whereas these IQ indexes were inversely associated with birth year of participants.

**Table 3:** Covariates in association with a change in intelligence quotient

Covariates	Multi variable model		
	Change in IQ	95% CI	*p*-value
*Covariates total intelligence quotient*			
Girls	− 2.57	− 5.32 to 0.17	0.07
Birth weight, + IQR	1.81	− 0.36 to 3.98	0.10
Gestational age, + IQR	0.17	− 1.67 to 2.00	0.86
Birth year, + 1 year	− 1.99	− 3.03 to − 0.96	0.0002
Peripheral cord insertion	1.31	− 1.39 to 4.00	0.34
Age at measurement, + 1 year	− 1.50	− 3.06 to 0.06	0.06
High parental educational level	6.99	4.19 to 9.79	< 0.0001
Neigbourhood household income, + IQR	− 0.42	− 2.10 to 1.25	0.62
Maternal smoking during pregnancy	− 1.46	− 6.21 to 3.29	0.55
Urban environment, + IQR	2.40	− 0.03 to 4.83	0.05
*Covariates verbal intelligence quotient*			
Girls	− 3.52	− 6.24 to − 0.79	0.01
Birth weight, + IQR	1.78	− 0.37 to 3.94	0.11
Gestational age, + IQR	− 0.13	− 1.88 to 1.62	0.88
Birth year, + 1 year	− 2.21	− 3.15 to − 1.26	< 0.0001
Peripheral cord insertion	0.87	− 1.79 to 3.54	0.52
Age at measurement, + 1 year	− 1.83	− 3.26 to − 0.40	0.01
High parental educational level	6.76	4.14 to 9.38	< 0.0001
Neigbourhood household income, + IQR	− 0.98	− 2.55 to 0.58	0.22
Maternal smoking during pregnancy	− 2.29	− 6.73 to 2.14	0.31
Urban environment, + IQR	1.54	− 0.77 to 3.84	0.19
*Covariates performance intelligence quotient*			
Girls	− 1.50	− 4.48 to 1.47	0.32
Birth weight, + IQR	0.99	− 1.31 to 3.28	0.40
Gestational age, +IQR	0.51	− 1.41 to 2.43	0.60
Birth year, + 1 year	− 1.18	− 2.27 to − 0.09	0.03
Peripheral cord insertion	0.56	− 2.28 to 3.39	0.70
Age at measurement, + 1 year	− 0.71	− 2.35 to 0.93	0.40
High parental educational level	5.57	2.65 to 8.49	0.0003
Neigbourhood household income, + IQR	0.14	− 1.61 to 1.88	0.88
Maternal smoking during pregnancy	− 0.09	− 5.04 to 4.86	0.91
Urban environment, + IQR	2.59	0.08 to 5.11	0.05

*CI* confidence interval, *IQR* interquartile range

### Placental mtDNA content in association with intelligence

Placental mtDNA content is associated with childhood intelligence 8 to 15 years later. Each doubling in mtDNA content is associated with a 2.0 point (95% CI 0.02 to 3.9; p = 0.05) higher in total IQ (Table 4). This association is driven by performance IQ. A doubling in placental mtDNA content is associated with a 2.3 point (95% CI 0.2 to 4.4; p = 0.03) higher performance IQ while we observed no association with verbal IQ. The association between placental mtDNA content and performance IQ remains significant when additional adjusting for behaviour problems (total CBCL T score). This is shown in Additional file 1: Table S2.

**Table 4:** Estimated change in intelligence quotient (IQ) for a doubling in mitochondrial DNA content

Intelligence quotient (IQ)	Change in IQ	95% CI	*p*-value
Total	1.98	0.02 to 3.93	0.05
Verbal	1.42	− 0.51 to 3.34	0.15
Performance	2.29	0.21 to 4.37	0.03

Adjusted for sex, gestational age, birth weight, birth year, zygosity and chorionicity, cord insertion, age at IQ measurement, indicators of socioeconomic status (parental education and neighbourhood household income), smoking during pregnancy and urban environment

*CI* confidence interval

**Fig. 1:**
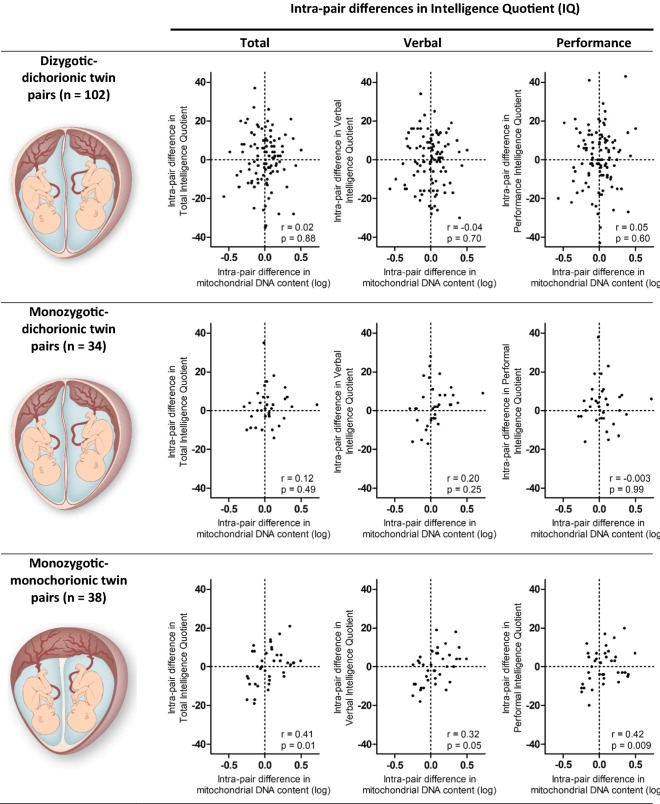
Correlation between intra-pair difference in mitochondrial DNA content in placental tissue and intra-pair differences in total, verbal and performance IQ in dizygotic (n = 102), monozygotic-dichorionic (n = 34) and monozygotic-monochorionic twin pairs (n = 38)

To unravel the complex interplay between early-life environmental and genetic risk factors and to improve the causal interpretation of our study we made use of the specific twin design. Pearson correlations between intra-pair differences in mtDNA content and intelligence quotient stratified by twin zygosity–chorionicity are shown in Fig. 1. Significant associations were observed in monozygotic-monochorionic twin pairs (n = 38) between placental mtDNA content and total (r = 0.41, p = 0.01), verbal IQ (r = 0.42, p = 0.009) and performance IQ (r = 0.32, p = 0.05). These results in monozygotic-monochorionic twins show further that the twin with the highest placental mtDNA content at birth is 1.9 times more likely (p = 0.05) to have the highest total IQ of the twins. This was not observed in dizygotic-dichorionic twin pairs (n = 102), neither in monozygotic-dichorionic twin pairs (n = 34).

### Placental mtDNA content and behaviour problems

No significant associations were observed between placental mtDNA content and total behaviour problems, neither for externalizing and internalizing problems (Table 5).

**Table 5:** Estimated change in behaviour problems (CBCL T score) for a doubling in mitochondrial DNA content

Behaviour problems	Change in CBCL T score	95% CI	*p*-value
Externalizing	0.07	− 1.36 to 1.51	0.92
Internalizing	− 0.43	− 1.95 to 1.08	0.58
Total	− 0.60	− 2.02 to 0.81	0.40

Adjusted for sex, gestational age, birth weight, birth year, zygosity and chorionicity, cord insertion, age at IQ measurement, indicators of socioeconomic status (parental education and neighbourhood household income), smoking during pregnancy and urban environment

*CI* confidence interval

## Discussion

Adaptive responses of the placenta may influence central nervous system development [1–3]. The key finding of our study was that placental mtDNA content is associated with child and adolescent IQ. Until now only studies in elderly noted an association between mtDNA content in blood and cognitive performance [11, 12]. This is the first study investigating placental mtDNA content and intelligence one decade later.

Our study benefits from its specific twin design to achieve a higher degree of causality from our observational data. Significant intra-pair differences between placental mtDNA content and intelligence were only observed in monozygotic-monochorionic twins. As monozygotic twins are genetically identical, the association between the intra-pair differences in mtDNA content and IQ must be due to the individual fetoplacental environment and specifically excludes the influence of genetic factors. Although monochorionic twins share only one placenta, intra-individual differences can be the result of an unequal degree of placental sharing [14]. In addition, placental sharing also enables differences between the individuals of the twin pair by allowing vascular connections between the circulation of the two fetuses and a higher prevalence of peripheral cord insertions [14]. In monozygotic-monochorionic twins unequal sharing of the placenta has been associated with a greater risk for birth weight discordance [24].

The significance of the fetal environment on intelligence in childhood is evident from twin studies, showing that twins score 4 point lower on an IQ test than singletons [25]. This difference is partially attributed to difference in intrauterine growth [25], nutrient deficiency, and shorter gestational length of twins [26]. Several studies show associations between mtDNA content in placental tissue and life-style or environmental parameters at birth. In the Belgian ENVIR*ON*AGE birth cohort, placental mtDNA content was 21.6% lower in mothers who smoked during pregnancy [27] and 17.4% lower for each 10 µg/m^3^ increment in PM_10_ exposure during the third trimester of pregnancy [10]. In a Spanish birth cohort (INMA), a positive association was observed between placental mtDNA content and birth weight [28]. Moreover, mtDNA content was postulated as a potential mediator of the association between prenatal air pollution exposure and birth weight [28]. Until now, potential consequences of decreased mtDNA content in early life on outcomes later in life were unknown.

The exact pathways underlying the association between mtDNA content in placental tissue and intelligence in childhood remains unclear. It is known that placental mitochondria play an important role in functioning of the placenta. Therefore, we assume that an insufficient performance of the placenta, as a results of low mtDNA content, might affect the brain of the developing fetus or alternatively—as indicated in previous indicated in experimental work—that the placenta reflects similar molecular signatures as in the fetal brain [1–3]. Besides its role in nutrient transfer and gas exchange, the placenta plays a key role in transfer and synthesis of neuroactive factors that are necessary for normal brain development [29, 30]. Neurotransmitter serotonin, essential for neurodevelopment, is not only provided by fetal and maternal sources but also synthetized by the placenta [31]. This is crucial for fetal brain development [1]. In addition, progesterone is synthesized by proteins located in the inner mitochondrial membrane of syncytiotrophoblast cells in the placenta [32]. Progesterone production by the placenta has a key role in providing precursors for the synthesis of neuroactive steroids in the fetal brain [33]. This highlights the importance of interactions between the placenta and the fetal brain [34]. Second, the most vulnerable early stages of fetal brain development depend on the placental transfer of maternal thyroid hormone [35]. Janssen et al. [27] observed that higher placental mtDNA content is associated with thyroid function more specifically with higher levels of free thyroid hormones in cord blood (FT_3_, FT_4_). A decrease in placental mtDNA content could be the result of hormone changes influencing optimal functioning of the placenta. Indirectly this could affect the development of the fetusus and their early brain.

Our findings in newborns parallels those in adult populations. Indeed, a significant reduction in mtDNA content is observed in brain tissue of patients with neurodegenerative diseases, such as Parkinsons’s and Alzheimer’s disease [36, 37]. The brain is susceptible to mitochondrial dysfunction due to its high energy demand [38] and because the brain, unlike most other tissues, depends exclusively on oxidative phosphorylation and glycolysis to create energy [39]. Furthermore neurotransmission depends on mitochondria not only for energy production but also for maintaining calcium homeostasis with the presynaptic terminal [40].

We observed a significant association between placental mtDNA content on performance IQ while no significant association was observed with verbal IQ. Verbal intelligence includes aspects like vocabulary and comprehension of language, while performance (nonverbal) intelligence includes matrix reasoning and picture completion. Our findings suggest that the regions of the brain associated with performance IQ are particularly affected by changes in placental mtDNA content during crucial periods of in utero development. Variations in placental function or other environmental insults during pregnancy such as maternal nutrition, might alter neurodevelopment in the fetus by promoting some brain regions over others [41].

The present study should be interpreted in the context of its limitations. First, an earlier study of Janssen et al. assessed within-placental variability of mtDNA content in a random subset of six placentas by comparing biopsies taken at four standardized sites across the middle region of the placenta [10]. mtDNA content within each placenta varied by a mean of 19.3% across the quadrants. To minimize the impact of within-placental variability in our study, biopsies used for mtDNA content assays were all taken at a fixed location. Second, it is a limitation that we have no information on cell composition of the placental biopsies. Third, we do not have information on vascular anastomoses for all twins. This may have an influence on placental sharing. Furthermore, our results show that placental mtDNA content is associated with IQ one decade later. Although its prospective nature, the present study is observational and cannot prove causation. However, the advantage of a twin study is that it can provide information on within-pair effects which increase causal interpretation. In this intra-pair analysis, we found in accordance with our primary analysis that the twin pair with the highest placental mtDNA content was 1.9 times more likely to have the highest score on total IQ 8 to 15 years later. Therefore, our study complies with important Hill criteria to establish causality. As our study is the first linking placental mitochondrial DNA content with childhood IQ others studies should confirm our association, preferentially also in a non-twin population to show the generalizability of the results.

For each doubling in placental mtDNA content we noted a 2.0 points increase in total intelligence and a 2.3 increase in performance IQ in childhood. Assuming causality, the magnitude of our estimates show that our molecular findings are relevant in a public health context. Indeed, although the impact on the population mean on the IQ might be moderate, children with a low placental mtDNA content will gravitate to the lower tail of the normal curve. Therefore, factors (both genetic, epigenetic and environmental) resulting in a lower placental mtDNA content might cause an inevitable “shift” in populations IQ. This has been demonstrated by Needleman for lead exposure during childhood, a greater proportion of children will gravitate to the lower tail of the normal curve [42, 43].

## Conclusions

Our study is the first to show an association between placental mitochondrial function as exemplified by placental mtDNA content and cognitive outcomes in 8–15 year olds. Our results emphasize the importance of the intrauterine environment on intelligence and the role of placental mitochondrial functions.

## Supplementary information

**Additional file 1: Text S1.** Mitochondrial and single copy-gene reaction mixture and PCR cycling conditions. **Table S1.** Primer sequences. **Table S2.** Estimated change in intelligence quotient (IQ) for a doubling in mitochondrial DNA content.

## Data Availability

The datasets used and/or analysed during the current study are available from the corresponding author on reasonable request.

## Abbreviations

CBCL: Child Behaviour Checklist
Cq: quantification cycle
C_T_: cycle threshold
DOHaD: The Developmental Origins of Health and Disease
EFPTS: The East Flanders Prospective Twin Survey
IQ: intelligence quotient
IQR: interquartile range
*MT*-*ND1*: mitochondrial encoded NADH dehydrogenase 1
mtDNA: mitochondrial DNA
*MTF3212/R3319*: mitochondrial forward primer from nucleotide 3212 and reverse primer from nucleotide 3319
PIQ: performance intelligence quotient
PM: particulate matter
qPCR: quantitative real-time polymerase chain reaction
*RPLP0*: acidic ribosomal phosphoprotein P0
TIQ: total intelligence quotient
VIQ: verbal intelligence quotient
WISC-R: Wechsler Intelligence Scale for Children-Revised
