# Supplementary material for: Placental mitochondrial DNA content is associated with childhood intelligence
Source: J Transl Med. 2019 Nov 8;17:361. doi: 10.1186/s12967-019-2105-y (PMC6839247; doi:10.1186/s12967-019-2105-y)
Supplement: Supplementary file 1 — Additional file 1: Text S1. Mitochondrial and single copy-gene reaction mixture and PCR cycling conditions. Table S1. Primer sequences. Table S2. Estimated change in intelligence quotient (IQ) for a doubling in mitochondrial DNA content. [file 12967_2019_2105_MOESM1_ESM.docx]

**Additional file**

**Placental alterations in mitochondrial DNA content predict childhood intelligence**

Esmée M Bijnens, Catherine Derom, Steven Weyers, Bram G Janssen, Evert Thiery,
Tim S Nawrot

**Table of contents**

Text S1 Mitochondrial and single copy-gene reaction mixture and PCR cycling conditions.

Table S1 Primer sequences

Table S2 Estimated change in intelligence quotient (IQ) for a doubling in mitochondrial DNA content.

**Text S1. Mitochondrial and single copy-gene reaction mixture and PCR cycling conditions.**

Extracted genomic DNA was diluted to a final concentration of 5 ng/μL in RNase free water prior to the qPCR runs. A 10 μL PCR reaction mixture contained QuantiTect SYBR® Green 1x (Qiagen, Venlo, The Netherlands), forward and reverse primer, and 2.5 µL of DNA sample. Primers sequences (Supplement table 1) were diluted to a final concentration of 300 nM into the master mix.

The thermal cycling profile was the same for all transcripts: 10 minutes at 95°C followed by 40 cycles of 15 seconds at 95°C for denaturation and 1 minute 10 seconds at 58°C for anneal­ing/extension. Amplification specificity and absence of primer dimers was confirmed by melting curve analysis at the end of each run (15 sec at 95°C, 15 sec at 60°C, 15 sec at 95°C).

| Table S1. Primer sequences | | |
| --- | --- | --- |
|  | Forward 5’-3’ | Reverse 5’-3’ |
| MTF3212/R3319 | CACCCAAGAACAGGGTTTGT | TGGCCATGGGTATGTTGTTAA |
| MT-ND1 | ATGGCCAACCTCCTACTCCT | CTACAACGTTGGGGCCTTT |
| RPLP0 | CAGCAAGTGGGAAGGTGTAATCC | CCCATTCTATCATCAACGGGTACAA |
| Abbreviations: *MTF3212/R3319,* mitochondrial forward primer from nucleotide 3212 and reverse primer from nucleotide 3319; *MT-ND1,* mito­chondrial encoded NADH dehydrogenase 1; *RPLP0,* acidic ribosomal phosphoprotein P0. | | |

| Table S2. Estimated change in intelligence quotient (IQ) for a doubling in mitochondrial DNA content. | | | |
| --- | --- | --- | --- |
| **Intelligence Quotient (IQ)** | **Change in IQ** | **95% CI** | ***P*-value** |
| Total | 1.93 | 0.02 to 3.83 | 0.05 |
| Verbal | 1.36 | - 0.53 to 3.25 | 0.16 |
| Performance | 2.26 | 0.24 to 4.27 | 0.03 |
| Adjusted for sex, gestational age, birth weight, birth year, zygosity and chorionicity, cord insertion, age at IQ measurement, behaviour problems (total CBCL T score, indicators of socioeconomic status (parental education and neighbourhood household income), smoking during pregnancy and urban environment. CI confidence interval | | | |
